# High Density Lipoprotein Reduces Blood Pressure and Protects Spontaneously Hypertensive Rats Against Myocardial Ischemia-Reperfusion Injury in an SR-BI Dependent Manner

**DOI:** 10.3389/fcvm.2022.825310

**Published:** 2022-03-21

**Authors:** Aishah Al-Jarallah, Fawzi Babiker

**Affiliations:** ^1^Department of Biochemistry, Faculty of Medicine, Kuwait University, Kuwait City, Kuwait; ^2^Department of Physiology, Faculty of Medicine, Kuwait University, Kuwait City, Kuwait

**Keywords:** high density lipoprotein, ischemia reperfusion injury, cardiac protection, hypertension, autophagy, inflammation

## Abstract

**Background:**

Hypertension is a key risk factor in the development of cardiovascular diseases. Elevation in blood pressure alters high density lipoprotein (HDL) function and composition. The exact role of HDL in cardiovascular complications observed in hypertension is however not clearly understood. HDL protected against myocardial ischemia/reperfusion (I/R) injury in normotensive rats. Nonetheless, it's not clear if restoration of HDL function and/or composition protects against myocardial I/R injury in spontaneously hypertensive rats (SHR).

**Objectives:**

In this study we tested the effect of HDL treatment on I/R injury in Wistar Kyoto rats (WKY) and SHR and investigated the possible underlying mechanism(s).

**Methods:**

HDL (900 ng/kg/min) or vehicle were continuously administered to 11-week old WKY and SHR for 1 week (chronic treatment). Blood pressure was measured before and after treatment. Hearts were subjected to I/R injury using a modified Langendorff system. Another set of rats were treated with HDL administered at reperfusion (acute treatment) in the presence or absence of scavenger receptor class B type-I (SR-BI) blocking antibody. Cardiac hemodynamics were computed and cardiac enzyme release and infarct size were measured. Total cholesterol (TC) and HDL-cholesterol (HDL-C) were enzymatically assayed. Markers of autophagy and inflammation were detected by immunoblotting and ELISA, respectively.

**Results:**

HDL treatment did not increase TC or HDL-C levels in SHR or WKY, yet it significantly (*P* < 0.01) reduced systolic and diastolic blood pressure in SHR. Chronic and acute HDL treatment significantly (*P* < 0.05) protected WKY and SHR against myocardial I/R injury. Chronic HDL treatment was significantly (*P* < 0.05) more protective in SHR whereas acute HDL treatment induced significantly (*P* < 0.05) greater protection in WKY. The extent of HDL induced protection was proportional to the expression levels of cardiac SR-BI and blockage of SR-BI completely abolished HDL mediated protection in SHR. Chronic HDL treatment significantly (*P* < 0.05) reduced markers of autophagy and inflammation in hypertensive rats.

**Conclusions:**

We demonstrate a novel anti-hypertensive and a cardioprotective effect of HDL against myocardial I/R injury in SHR, the magnitude of which is directly related to the expression levels of cardiac SR-BI. Mechanistically, chronic HDL treatment protected SHR hearts by reducing autophagy and inflammation.

## Introduction

Hypertension is a leading cause of premature deaths. In 2015, elevated systolic blood pressure (SBP) claimed the lives of 8.5 million individuals worldwide (1–3). It represents an ever growing health condition that imposes a huge financial burden on the economy of the affected countries. The number of hypertensive individuals doubled from 1990 to 2019 to be 652 million males and 626 million females (2) and the cost of hypertension per country has been estimated to be several dozen billion Int$ (3). In addition, hypertension is a major risk factor of cardiovascular diseases (CVD), whereby increased blood pressure attributes to 47% of coronary heart disease (CHD) cases (4, 5). Moreover, blood pressure lowering is an effective strategy in reducing blood pressure related deaths (6–9). Control randomized clinical trials demonstrated that patients at high cardiovascular risk or patients with established CVD benefit from blood pressure lowering treatments whether they were normotensive or hypertensive (6–11). In addition an inverse relationship exists between HDL levels and the risk of CHD (12–16). Low HDL cholesterol (HDL-C) and hypertension are symptoms of metabolic syndrome and both are important predictors of acute myocardial infarction (17). HDL-C levels are inversely associated with the risk of hypertension (18, 19) and a combination of high-normal blood pressure with low HDL-C was associated with increased mortality (20). Reduced HDL-C levels and impaired HDL synthesis and turnover were reported in patients with mild hypertension (21). Furthermore, increased blood pressure eliminated the protective effects of HDL against CHD and stroke (9). HDL function and composition were altered by hypertension and anti-hypertensive drugs. Reduced contents of HDL phospholipids and changed phospholipid composition represented as a lower ratio of phosphatidylcholine and higher relative levels of lysophosphatidylcholine, sphingomyelin and phosphatidylethanolamine were reported in patients with hypertension (22). The exact relationship between HDL and hypertension is however not clearly understood.

Deteriorated cardiac functions (23–26) and enhanced cardiac autophagy (27–29) were reported in experimental models of hypertension. Compared to normotensive controls, hearts from experimental models of hypertension were resistant to protection induced by ischemic post-conditioning (30–32), erythropoietin (33), helium (34), and short-term infusion of captopril (35). *In vivo* and *ex vivo* administration of HDL or HDL components protected rodent hearts from ischemia reperfusion (I/R) injury (36). Acting on cardiomyocytes, endothelial cells and leukocytes, HDL induced cardioprotection involved simultaneous inhibition of the damaging effects of I/R injury and activation of internal protective responses including the activation of the reperfusion injury salvage (RISK) (37) and the survivor activating factor enhancement (SAFE) pathways and subsequent inhibition of mitochondrial permeability transition pore (mPTP) opening (38). In addition, HDL stimulated the release of vasoactive (37) and cardioprotective compounds (39). Nonetheless it's not clear if HDL can protect hearts from hypertensive rodents against myocardial I/R injury. We therefore sought to test the effect of HDL on I/R injury in normotensive, Wistar Koyoto rats (WKY), and spontaneously hypertensive rats (SHR) and examine the involvement of autophagy and inflammation as possible mechanisms of HDL mediated effects. We demonstrate that HDL treatment reduces SBP and diastolic blood pressure (DBP) in SHR. Chronic and acute HDL treatments protected WKY and SHR against myocardial I/R injury yet, to different extents. Chronic HDL treatment induced greater protection in SHR, while acute HDL treatment was more protective in WKY. Interestingly the magnitude of HDL protection in WKY and SHR was proportional to the expression levels of cardiac SR-BI in these rats. HDL was more protective against myocardial I/R injury in rats expressing greater levels of cardiac SR-BI. Blockage of SR-BI completely abrogated HDL mediated protection against I/R injury in SHR. HDL treatment reduced autophagy markers beclin, microtubule-associated protein 1 light chain 3 (LC-3) B and autophagy regulated gene (Atg)-12 and tumor necroses factor-α (TNF-α) levels in SHR suggesting that HDL mediated cardiac protection in hypertensive rats involves simultaneous attenuation of multiple detrimental pathways including autophagy and inflammation. Our data emphasize a multifaceted role of HDL in protecting hypertensive rats against myocardial I/R injury by virtue of its systemic (blood pressure lowering) and/or local (attenuation of inflammation and autophagy) effects.

## Materials and Methods

### Materials

All materials were purchased from Sigma Aldrich (Munich, Germany) unless stated otherwise.

### Animals and Instrumentation

In experiments testing the effect of chronic HDL treatment on I/R injury, 11-week old male WKY (334 ± 29 g) and SHR (282 ± 20 g) were used. Animals were kept under internationally accepted conditions in the Animal Resource Center, Faculty of Medicine, Kuwait University and had free access to food and water. All procedures that involved rats were approved by the Health Sciences Research Ethics Committee. The rats were divided into four groups (*n* = 12 rats per group): (1) WKY-vehicle; (2) WKY-HDL; (3) SHR-vehicle; (4) SHR-HDL. HDL (900 ng protein/kg/min) or phosphate-buffered saline (PBS) (as a vehicle) were continuously administered using Alzet osmotic minipumps implanted subcutaneously into the back of the rats. This dosage was selected based on previously reported data by Lin et al. (40). One week later, rats were sacrificed, plasma samples were collected and cardiac response to I/R injury was examined. Blood pressure was measured prior and post-implantation or at week 12 in non-implanted rats using CODA High Throughput System with 4 activated channels (41). SBP ≥160 mmHg was used as a cutoff value for hypertension. Rats that did not reach the cutoff value were excluded from the study. In experiments testing the effects of acute HDL treatment and the involvement of SR-BI, 12-week old WKY and SHR (*n* = 30) were used. Finally, a distinct cohort of 12-week old WKY and SHR (*n* = 22) was set to test the effect of 3-methyladenine (3-MA).

Heart cannulation and perfusion was performed as described previously (42). Briefly, isolated hearts were perfused retrogradely with freshly prepared Krebs-Hensleit buffer, pH 7.35–7.45 at 37.0 ± 0.5°C and gassed with CO_2_ (5%) and O_2_ (95%). Regional ischemia was induced by occluding left anterior descending (LAD) coronary artery for 30 min. The success of ischemia induction was evaluated online at the onset of ischemia by the immediate drop in the coronary flow. Two rats were excluded from the study because of left ventricular (LV) fibrillation. Preload was kept constant at 6 mmHg under basal controlled conditions and perfusion pressure (PP) at 50 mmHg throughout the experimental procedure in all protocols. The perfusion pressure was measured downstream from a branch of the aortic cannula using a “statham pressure transducer” (P23 Db). Constant PP was ensured electronically by means of the perfusion assembly [“Module PPCM type 671 (Hugo Sachs Elektronik- Harvard ApparatusGmbH, Germany”)], an effective system for the accurate adjustment of PP between 5 and 150 mmHg with ±1 mmHg accuracy level.

### Study Protocol and Study Groups

**Figure 1:**
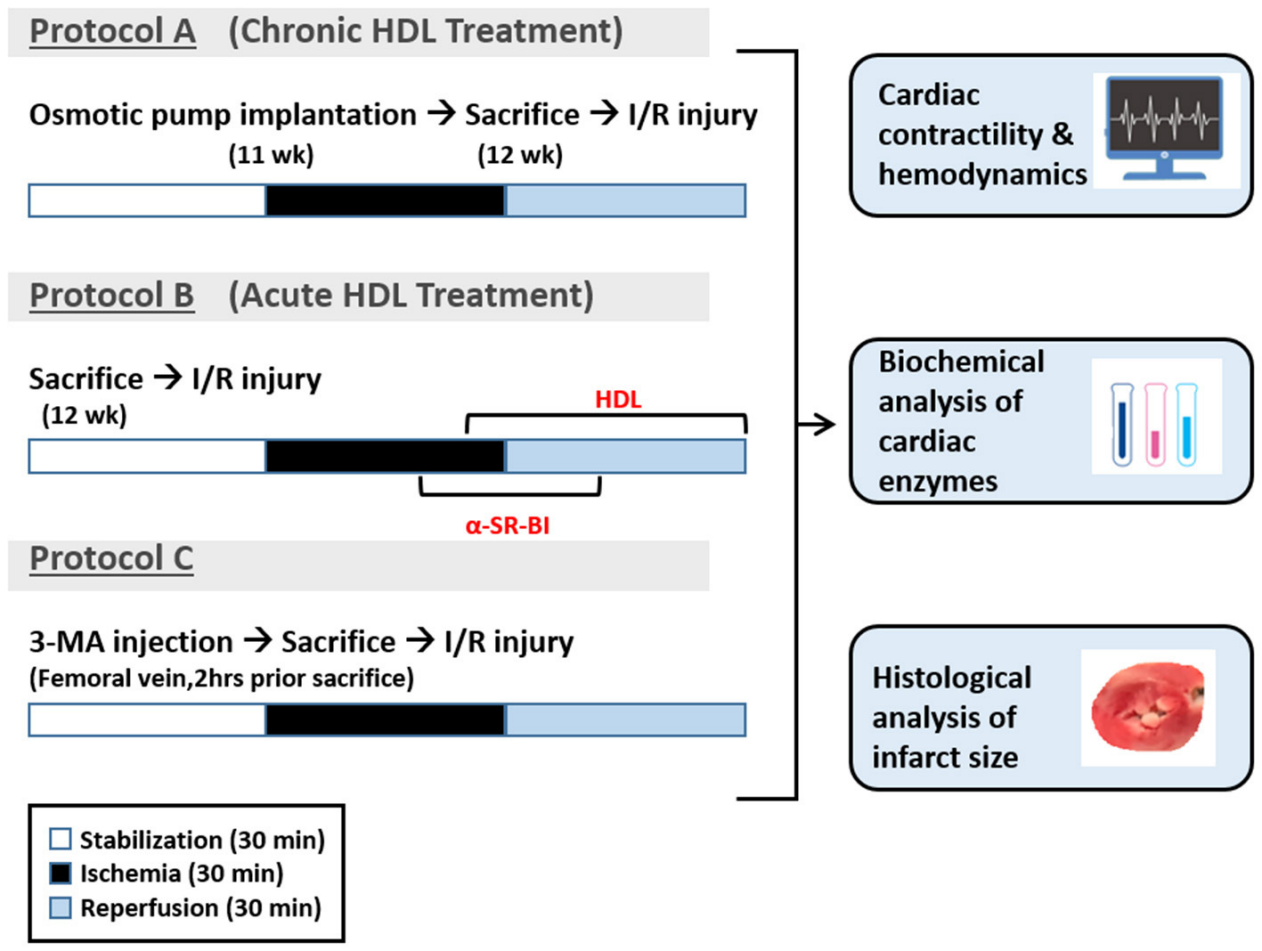
Experimental protocols used in the study. Eleven week old WKY and SHR were implanted with vehicle control or HDL containing osmotic pumps. One week later, rats were sacrificed and subjected to ischemia/reperfusion injury (I/R) with 30 min of stabilization followed by 30 min of ischemia and 30 min of reperfusion (protocol A). A group of untreated WKY and SHR were sacrificed and subjected to I/R injury. SR-BI blocking antibody was administered at 20 min of ischemia. Ten minutes later, hearts were perfused with HDL (400 μg) in the presence of SR-BI blocking antibody for the remaining of the 30 min the reperfusion period (protocol B). Another group of untreated WKY and SHR were injected with 3-MA (30 mg/Kg) into the femoral vein. Two hours later, rats were sacrificed and subjected to 30 min of ischemia followed by 30 min of reperfusion (protocol C).

All hearts were subjected to 30 min of ischemia produced by LAD coronary artery occlusion. The LAD coronary artery was encircled by a snare at ~0.5 cm below the atrioventricular groove, and a small rigid plastic tube was positioned between the heart and the snare to ensure complete occlusion of the coronary artery. Hearts were then reperfused for 30 min without any additions to the perfusion buffer (Figure 1, protocol A). In experiments testing the involvement of SR-BI in HDL mediated cardiac protection, hearts isolated from WKY and SHR were infused with SR-BI blocking antibody (Novus Biologicals, CO, USA, NB400-113, 1:100) during the last 10 min of ischemia, then perfused with or without HDL (acute treatment, 400 μg protein) in the presence of the antibody for the remaining 30 min of the reperfusion period, using a total volume of 150 ml of the perfusion buffer (Figure 1, protocol B). The role of autophagy in I/R injury was tested by the administration of 3-MA (30 mg/Kg) into the femoral vein 2 h prior to sacrifice followed by 30 min of ischemia and 30 min of reperfusion without any additions to the perfusion buffer (Figure 1, protocol C).

### Data Collection and Processing

LV function was evaluated by the assessment of LV end diastolic pressure (LVEDP) and LV developed pressure (DPmax), cardiac contractility was monitored by heart contractility index values (±dP/dt), while coronary-vascular dynamics were evaluated by the assessment of the coronary flow (CF) and coronary vascular resistance (CVR). Cardiovascular functions were measured as previously described (42). Briefly, a water-filled latex balloon was placed and secured in the LV cavity. The balloon was attached to a pressure transducer and a “DC-Bridge amplifier (DC-BA)” with a pressure module (DC-BA type 660, Hugo-Sachs Electronik, Germany) and interfaced to a personal computer for on-line monitoring of DPmax. LV developed pressure was derived from online acquisition of LV end systolic pressure (LVESP) using Max-Min module (Number MMM type 668, Hugo Sachs Elektronik-Harvard ApparatusGmbH, Germany) which has the ability to convert the output from DC bridge amplifier to DPmax by subtracting LVEDP from the LVESP.

Coronary flow was continuously measured using an electromagnetic flow probe attached to the inflow of the aortic cannula interfaced to a personal computer. The continuous monitoring of the CF in ml/min was digitally monitored using software developed specifically for this purpose and was manually verified by the timed collection of coronary effluent. The CVR and hemodynamics data were determined every 10 s using an on-line data acquisition program (Isoheart software V 1.524-S, Hugo-Sachs Electronik, Germany). By the end of each experiment, hearts were weighed then snap frozen in liquid nitrogen then stored in −80°C. The tibia was isolated and its length was measured with ruler and the heart weight was expressed relative to the tibia length.

### Evaluation of Cardiac Injury by Measurements of Infarct Size and Cardiac Enzyme Levels

The infarct size was determined by triphenyltetrazolium chloride (TTC) staining as described previously (32). Each heart was sliced into 4–5 pieces along the long axis. The slices were then incubated for 15 min in 1% TTC solution in isotonic phosphate buffer (pH 7.4) at 37°C and fixed in 4% formaldehyde. Images were taken using a Samsung camera. Red and pale unstained areas of every slice were indicated manually on the image using Leica ImageJ (Image J, Wayne Rasb and National Institute of Health, USA). The percentage infarct area was calculated relative to total LV area. Cardiomyocyte injury was evaluated by measuring creatine kinase (CK), or troponin release in the coronary effluent during the reperfusion period as previously described (43). Briefly, CK was measured using a CK specific kit (catalog # 442635, Beckman Coulter, CA, USA) with an analytical range of 5–1,200 IU/L and ran using UniCel^®^ DXC 800 Synchron Access Clinical Systems (Beckman Coulter, CA, USA). Troponin was measured using troponin specific kit (catalog # B52699, Beckman Coulter, CA, USA) with a quantification limit of 5.6 pg/ml at 10% CV and ran using UniCel^®^ DXI 800 Access Immunoassay System (Beckman Coulter, CA, USA).

### Immunoblotting

Heart homogenates were prepared using a buffer containing 0.2 × PBS, 0.1 % triton-X 100, 1 × phosphatase inhibitor and 1 × protease inhibitor cocktail then centrifuged at 14,000 rpm in a bench top minicentrifuge for 10 min at 4°C. Membrane fractions were prepared by homogenizing hearts on ice for 3 min in 20 mM Tris-HCl, pH 7.5 containing 2 mM MgCl_2_, 0.25 M sucrose, and 1 × protease inhibitors. Homogenates were centrifuged at 3,000 × g for 10 min at 4°C and supernatants were subjected to another centrifugation step at 100,000 × g for 1 h at 4°C. The pellet was suspended in 50 mM Tris-HCl, pH 7.5 containing 1 × protease inhibitors cocktail and 0.1% sodium dodecyle sulfate. Protein concentration from total homogenates and membrane fractions was measured using the BCA-protein determination kit (Themoscientific, Ottawa, Ontario, CA) and samples were aliquoted and stored at −80°C for further analysis. After boiling, samples (50 μg protein) were subjected to SDS-PAGE and PVDF membranes were immunoblotted with anti-beclin-1, LC3B, Atg-3, Atg-5, Atg-7, Atg-12 (Cell Signaling, MA, USA), or anti-SR-BI (Abcam, MA, USA) overnight, followed by HRP-conjugated donkey anti-rabbit antibody (Jackson ImmunoResearch, PA, USA). Bands were detected using Super Signal Western Pico chemiluminescence Substrate (Thermoscientific, Ottawa, Ontario, CA) and quantified using Biorad gel doc RX System (BioRad, CA, USA).

### Measurements of Plasma Cholesterol

Plasma total cholesterol (TC) and HDL cholesterol (HDL-C) were measured enzymatically using commercially available kit (Abcam, MA, USA) following the manufacturer instructions.

### Measurements of TNF-α and IL-6 Levels

Total heart homogenates were assayed suing TNF-α and IL-6 ELISA kits (Thermosceinfic, Ottawa, Ontario, CA) according to manufacturer instructions.

### Statistical Analysis

Multiple comparisons were evaluated using two-way analysis of variance (ANOVA). In cases of statistical significance, the *post-hoc* analysis using LSD test was performed (SPSS). Student's *T*-Test was used to assess the significance in molecular experiments (Microsoft Excel). Data were considered statistically significant at *P* < 0.05. Data are presented as mean ± standard error of the mean (SEM) of “*n*” number of experiments.

## Results

### Effects of HDL Treatment on Blood Pressure and Plasma Cholesterol Levels

**Figure 2:**
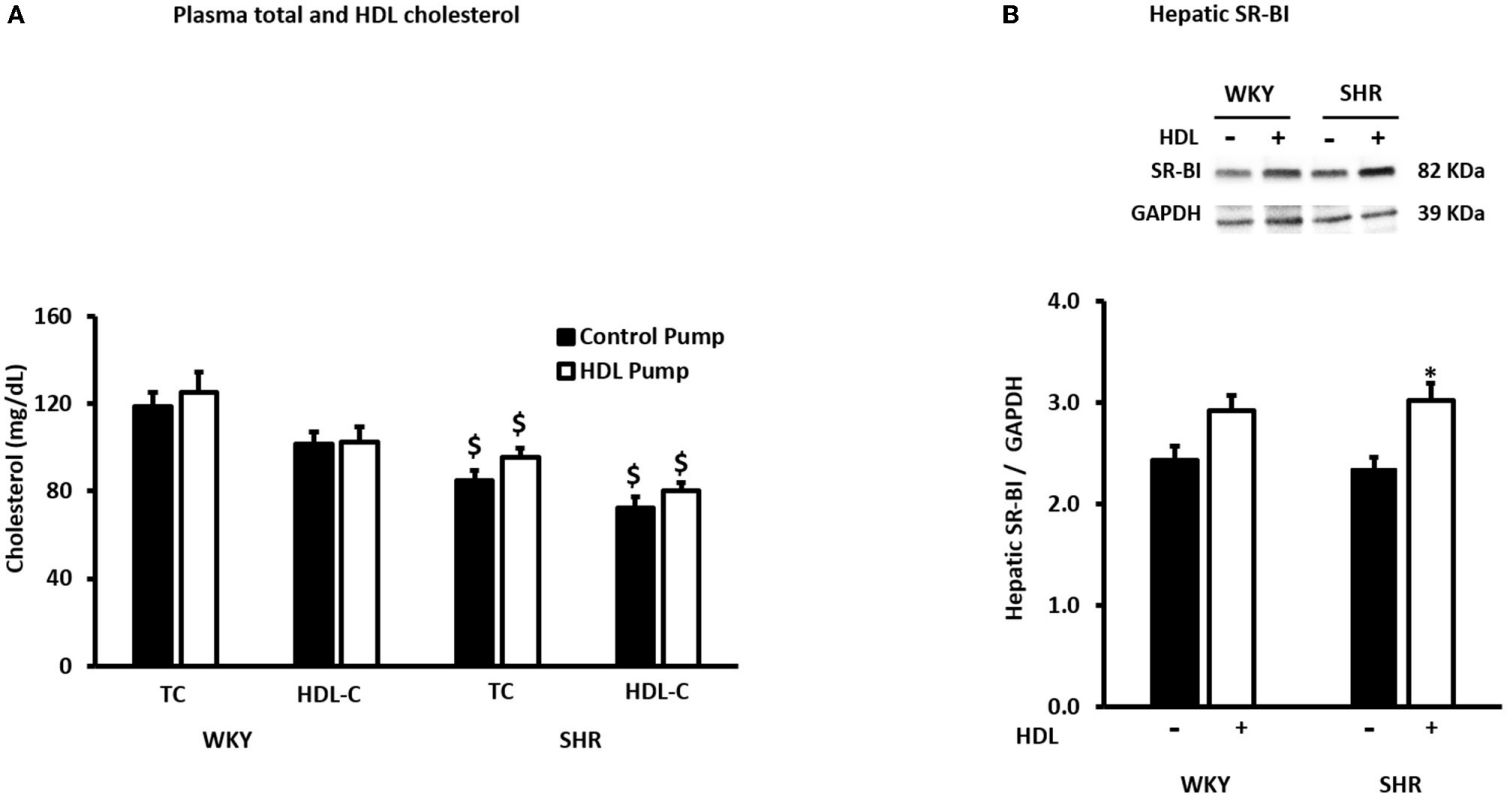
Effects of HDL treatment on plasma cholesterol and hepatic SR-BI expression in WKY and SHR. Plasma total and HDL cholesterol levels **(A)** and hepatic SR-BI expression **(B)** in WKY and SHR treated with or without HDL. Data are mean ± SEM, ^$^*P* < 0.05 vs. the same parameter and treatment group in WKY, *n* = 7–10, *P < 0.05 vs. untreated animals of the same genotype, *n* = 5–6.

SHR exhibited significantly higher (*P* < 0.01) SBP and DBP than WKY (Table 1). HDL treatment significantly (*P* < 0.01) reduced SBP and DBP in SHR by 20% and 23% relative to preimplantation levels, respectively, and by 20% (SBP) and 27% (DBP) relative to SHR implanted with the vehicle pump (Table 1). SBP and DBP in HDL-treated SHR were not significantly (*P* > 0.05) different from WKY (Table 1). HDL treatment did not have a significant (*P* > 0.05) effect on heart to tibia length ratio in WKY or SHR (Supplementary Figure 1). Finally, SHR demonstrated significantly (*P* < 0.05) lower total cholesterol and HDL-C than WKY and HDL treatment did not significantly (*P* > 0.05) alter plasma total cholesterol or HDL-C in SHR or WKY (Figure 2A). The lack of increase in HDL-C in HDL treated rats maybe explained by the trend toward an increase (in WKY) and the statistically significant (*P* < 0.05) increase (SHR) in hepatic SR-BI protein levels (Figure 2B) suggesting enhanced clearance of HDL in HDL treated rats.

**Table 1:** Blood pressure measurements of WKY and SHR treated with or without HDL.

	**Control**				**HDL**			
	**Pre-implantation**		**Post-implantation**		**Pre-implantation**		**Post-implantation**	
	**SBP**	**DBP**	**SBP**	**DBP**	**SBP**	**DBP**	**SBP**	**DBP**
**WKY**								
Mean ± SEM (mmHg)	115 ± 2	72 ± 1.9	107 ± 4	70 ± 3.5	130 ± 3	88 ± 2.5	124 ± 6.8	83 ± 6
**SHR**								
Mean ± SEM (mmHg)	174 ± 4.5^#^	120 ± 6^#^	169 ± 6	117 ± 6	169 ± 4^#^	111 ± 5^#^	136 ± 4.4*^$^	85 ± 4*^$^

^#^*P <0.01 vs. WKY*.

^*^P <0.01 vs. pre-implantation.

^$^*P <0.01 vs. control SHR*.

### HDL Protects WKY and SHR Against Myocardial I/R Injury to Different Extents

**Figure 3:**
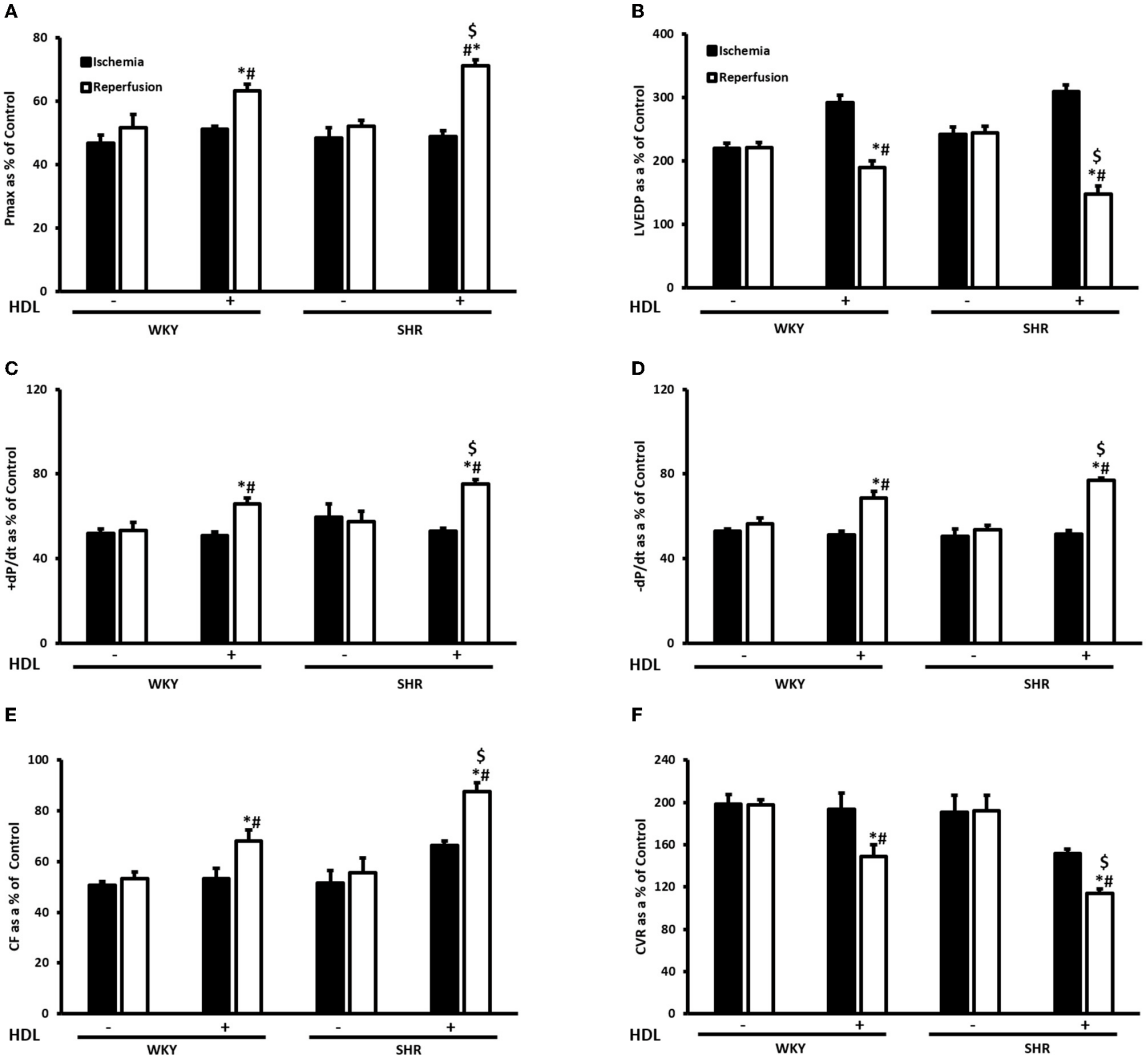
Effect of chronic HDL treatment on I/R injury in WKY and SHR. Post-ischemic recovery parameters of cardiac functions including DPmax **(A)**, LVEDP **(B)**, cardiac contractility **(C,D)**, CF **(E)**, and CVR **(F)**. Data were computed at 30 min of reperfusion and expressed as means ± SEM. DPmax, maximum developed pressure; LVEDP, left ventricular end diastolic pressure; CF, coronary flow; CVR, coronary vascular resistance. *P < 0.5 compared to ischemic period, ^#^*P* < 0.5 compared to control pump, ^$^*P* < 0.05 vs. HDL treated WKY, *n* = 6–10 rats per group.

**Figure 4:**
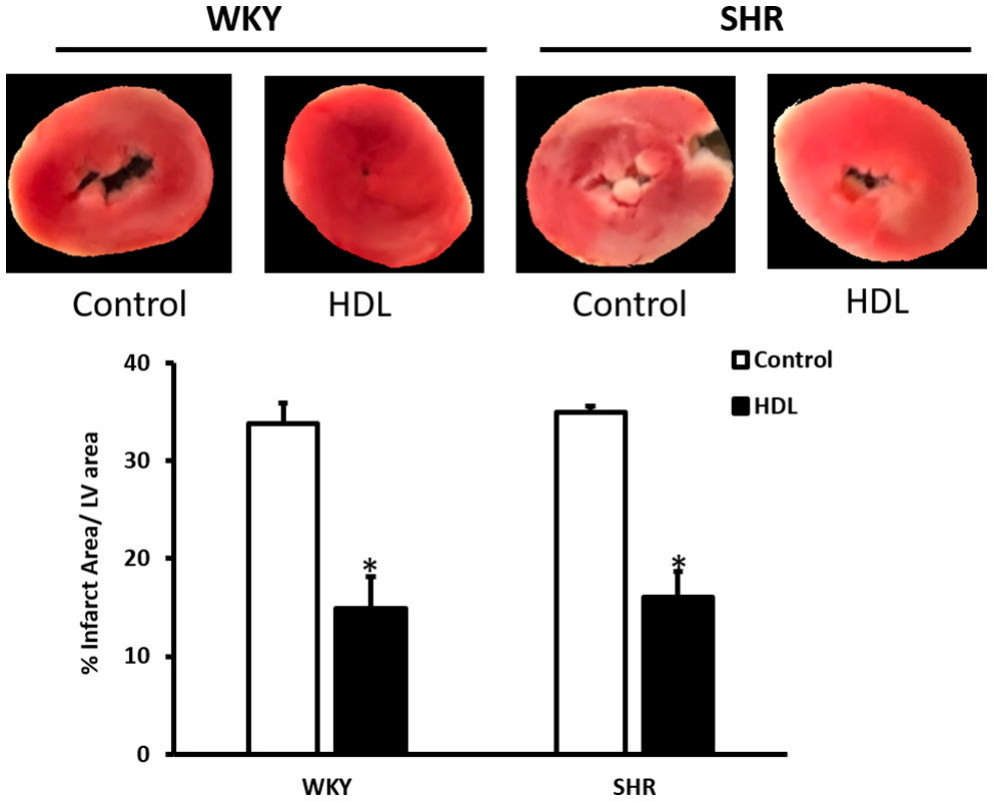
HDL treatment reduces infarct size in WKY and SHR. WKY and SHR rats were treated with or without HDL and subjected to I/R injury *ex-vivo*. Infarct size was quantified in TTC stained sections. Data are expressed as means ± SEM, *P < 0.01, *n* = 3–5 per treatment.

Hearts from WKY and SHR treated with or without HDL were isolated and subjected to I/R injury *ex-vivo*. Cardiac contractility and hemodynamics were recorded and the extent of ischemic damage was assessed by cardiac enzyme release and measurements of infarct size. Hearts from normotensive rats treated with HDL (chronic treatment) demonstrated significant (*P* < 0.05) improvements in DPmax and LV pressure (Figures 3A,B), cardiac contractility (Figures 3C,D) and cardiac hemodynamics (Figures 3E,F) during reperfusion compared to ischemic period and relative to untreated controls. Similarly, chronic HDL treatment of SHR significantly (*P* < 0.05) improved DPmax, LV pressure, cardiac hemodynamics, and contractility relative to the ischemic period and relative to SHR controls (Figures 3A–F). HDL however, demonstrated significantly (*P* < 0.05) enhanced protection on cardiac contractility and hemodynamics in SHR relative to WKY (Figures 3A–F). Moreover, measurements of cardiac enzymes revealed significantly (*P* < 0.05) higher levels of CK in control SHR relative to control WKY during periods of ischemia and reperfusion (Table 2), possibly suggesting enhanced susceptibility of hearts from SHR to cardiac damage. Chronic HDL treatment significantly reduced (*P* < 0.05) CK release in WKY and SHR by 46% and 69%, respectively (Table 2). In addition, chronic HDL treatment significantly (*P* < 0.05) reduced infarct size in WKY and SHR (Figure 4). Collectively this data suggest that chronic, *in vivo*, HDL treatment protects WKY and SHR against I/R injury. The extent of HDL-mediated protection however, appears to be different between normotensive and hypertensive rats. The observed differences in HDL mediated cardiac protection may possibly suggest differences in HDL signaling between WKY and SHR.

**Table 2:** Effect of chronic HDL treatment on creatine kinase release in response to I/R injury.

**CK (IU/L)**				
	**Control**		**HDL**	
	**Ischemia**	**Reperfusion**	**Ischemia**	**Reperfusion**
**WKY**	3.5 ± 0.54	3.2 ± 1.1	6.2 ±1.8	3.3 ±1.1
**SHR**	10.5 ± 2.5^$^	27.76 ± 0.5^#$^	12.3 ± 3.1	3.8 ± 0.8^#*^

^$^*P < 0.05 vs. WKY*.

^#^*P < 0.05 vs. ischemia*.

*P < 0.05 vs. untreated controls.

### HDL Requires SR-BI for Protection Against Myocardial I/R Injury

**Figure 5:**
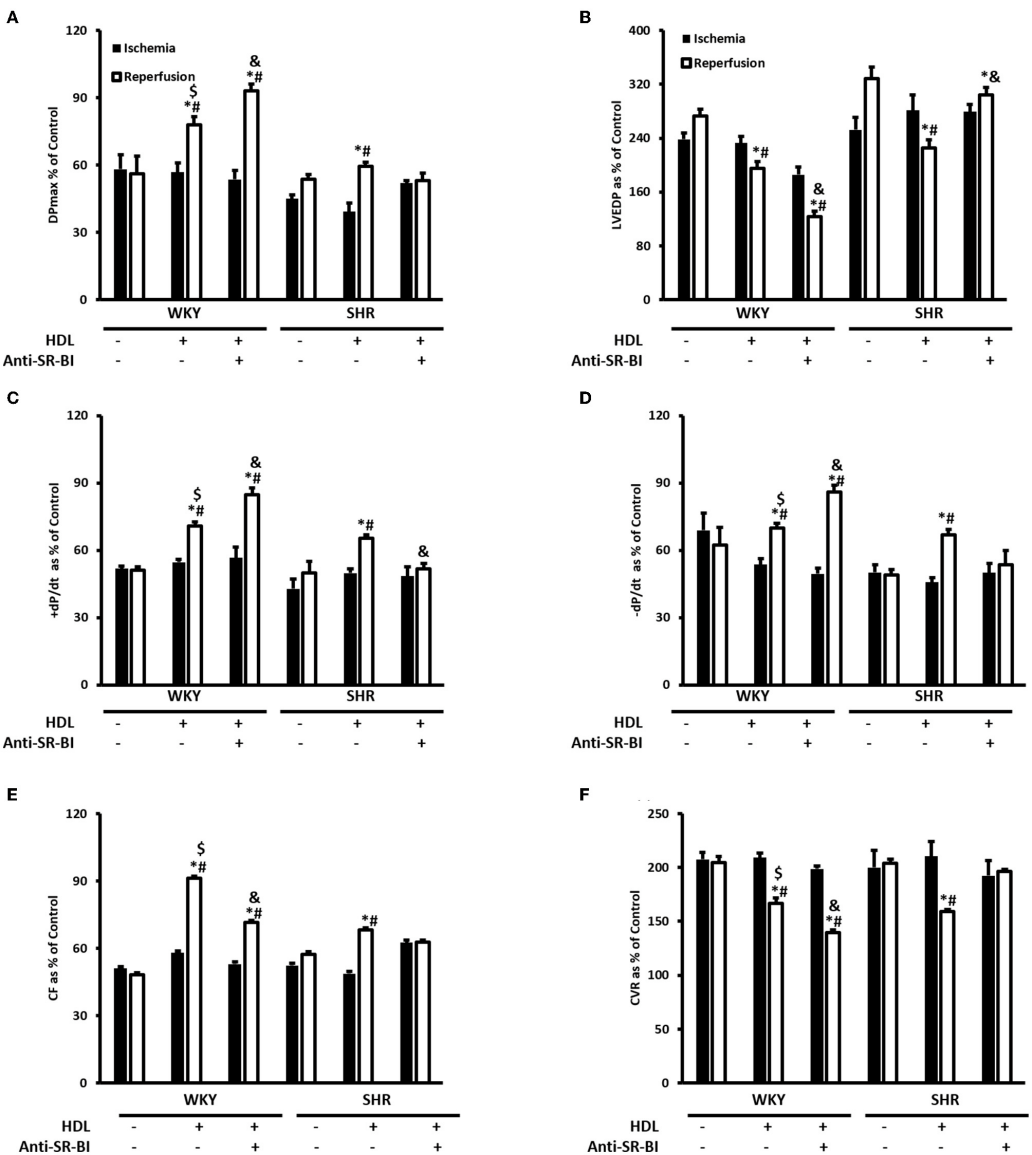
The role of SR-BI in HDL mediated cardiac protection against I/R injury in WKY and SHR. Post-ischemic recovery parameters of cardiac functions including DPmax **(A)**, LVEDP **(B)**, cardiac contractility **(C,D)**, coronary hemodynamics **(E,F)**. Data were computed with no addition control, with HDL (400 μg) administered at reperfusion (acute treatment) in the presence or absence of SR-BI blocking antibody and expressed as means ± SEM. DPmax, maximum developed pressure; LVEDP, left ventricular end diastolic pressure; CF, coronary flow; CVR, coronary vascular resistance; *P < 0.5 compared to ischemic period. ^#^*P* < 0.5 compared to no addition control, ^$^*P* < 0.05 relative to HDL treated SHR, ^&^*P* < 0.05 vs. HDL treatment of the same genotype, *n* = 3–8 rats per group.

We have tested the involvement of the HDL receptor, SR-BI in HDL mediated cardiac protection in WKY and SHR using SR-BI blocking antibody (Figure 5). In these experiments HDL was administered at reperfusion (acute treatment) in the presence or absence of SR-BI blocking antibody. HDL administration at reperfusion significantly (*P* < 0.05) improved cardiac contractility and hemodynamics in WKY and SHR (Figures 5A–F). HDL however, was more protective in WKY than SHR when administered at reperfusion as indicated by the significant (*P* < 0.05) increase in DPmax (Figure 5A), ±dp/dt (Figures 5C,D) and CF (Figure 5E) and the significant (*P* < 0.05) decrease in CVR (Figure 5F).

Furthermore, the administration of HDL at reperfusion in the presence of SR-BI blocking antibody significantly (*P* < 0.05) increased DPmax, cardiac contractility (+dP/dt and –dP/dt) and CF (Figures 5A,C–E) and significantly (*P* < 0.05) reduced LVEDP and CVR relative to ischemia and relative to untreated controls in WKY (Figures 5B,F). Moreover, HDL treatment in the presence of SR-BI blocking antibody in WKY, significantly (*P* < 0.05) increased DPmax and CVR (Figures 5A,F) and significantly (*P* < 0.05) reduced LVEDP, CF relative to HDL-treated rats (Figures 5B,E). This suggests blockage of SR-BI did not abolish HDL induced cardiac protection in WKY, rather it reduced it. In SHR however, HDL treatment in the presence of SR-BI blocking antibody did not result in any significant improvements in cardiac contractility, hemodynamics or ventricular pressure relative to ischemia or relative to the untreated controls (Figures 5A–F), suggesting the absolute requirement of SR-BI in HDL mediated cardiac protection in SHR.

Together, this data suggest that while SR-BI plays an indispensable role in mediating HDL induced cardiac protection in SHR, this however, does not seem to be the case in normotensive rats. In WKY HDL remained protective, albeit to a lesser extent than HDL alone, in the presence of SR-BI blocking antibody. The residual HDL mediated cardiac protection in WKY in the presence of SR-BI blocking antibody, possibly suggests the presence of alternative or additional pathways or mechanisms by which HDL protects against cardiac I/R injury. Interestingly, the magnitude of HDL induced cardiac protection in WKY and SHR varied between the chronic and acute treatments. In chronic treatment HDL was more protective in SHR (Figure 3), however in the acute treatment, HDL was more protective in WKY (Figure 5). The finding that SR-BI is required for HDL mediated cardiac protection in WKY and SHR may implicate differences in SR-BI mediated signaling in HDL induced protection against myocardial I/R injury.

**Figure 6:**
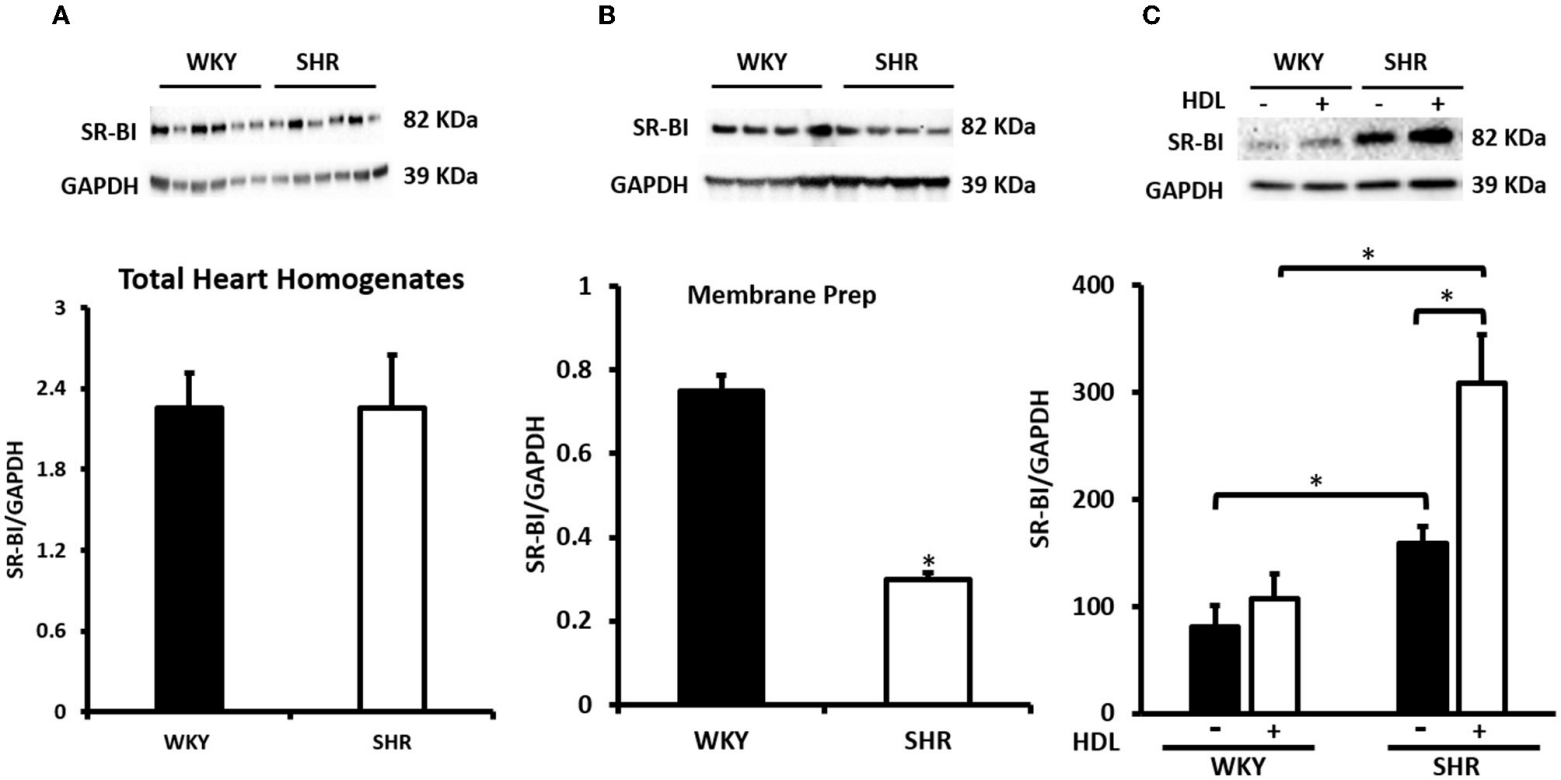
Cardiac SR-BI Expression in WKY and SHR. Total heart homogenates **(A)** and membrane preparations **(B)** from WKY and SHR and total heart homogenates from WKY and SHR implanted with vehicle or HDL containing osmotic pumps **(C)** were subjected to immunoblotting against SR-BI and GAPDH as a loading control. Data are expressed as means ± SEM, *P < 0.05, *n* = 5–6 rats per group.

We have therefore examined the expression levels of SR-BI in WKY and SHR subjected to chronic or acute HDL treatment (Figure 6). There was no significant difference in basal SR-BI protein levels in total heart homogenates from WKY and SHR (Figure 6A). Nonetheless, WKY expressed significantly (*P* < 0.01) higher levels of cardiac SR-BI than SHR in membrane fractions (Figure 6B) suggesting differences in SR-BI localization between WKY and SHR. Finally, total heart homogenates from WKY and SHR treated *in vivo* (chronically) with or without HDL were examined (Figure 6C). Significantly (*P* < 0.05) higher levels of cardiac SR-BI were detected in vehicle treated SHR compared to vehicle treated WKY. Chronic HDL treatment significantly (*P* < 0.05) increased cardiac SR-BI expression in SHR (Figure 6). These changes in receptor expression levels in response to HDL were not however observed in WKY. Collectively this data suggest the possibility that the observed differences in the magnitude of HDL induced protection between WKY and SHR in response to chronic or acute treatment could be attributed to differences in SR-BI expression levels. HDL induced greater protection against I/R injury in hearts expressing greater levels of SR-BI.

### HDL Attenuates Autophagy and Inflammation in SHR

**Figure 7:**
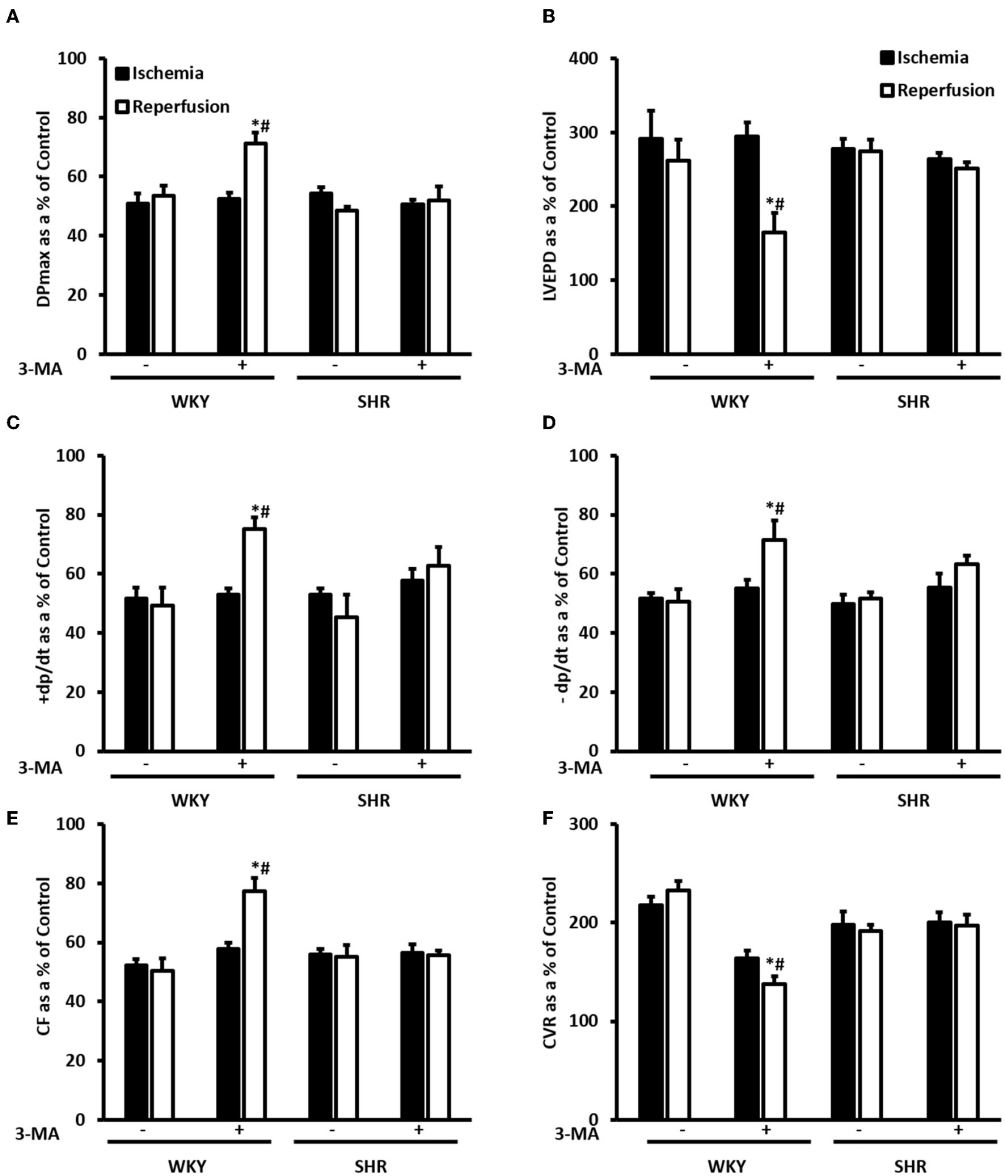
Treatment with 3-MA protects WKY but not SHR against I/R injury. Post-ischemic recovery parameters of cardiac functions including DPmax **(A)**, LVEDP **(B)**, coronary hemodynamics **(C,D)**, cardiac contractility **(E,F)**. Data were computed with no addition control, with 3-MA (30 mg/Kg) administered into the femoral vein 2 h prior to sacrifice. Data are expressed as means ± SEM. DPmax, maximum developed pressure; LVEDP, left ventricular end diastolic pressure; CF, coronary flow; CVR, coronary vascular resistance; 3-MA, 3-methyladenine; *P < 0.5 compared to ischemic period. ^#^*P* < 0.5 compared to no addition control, *n* = 4–7.

**Figure 8:**
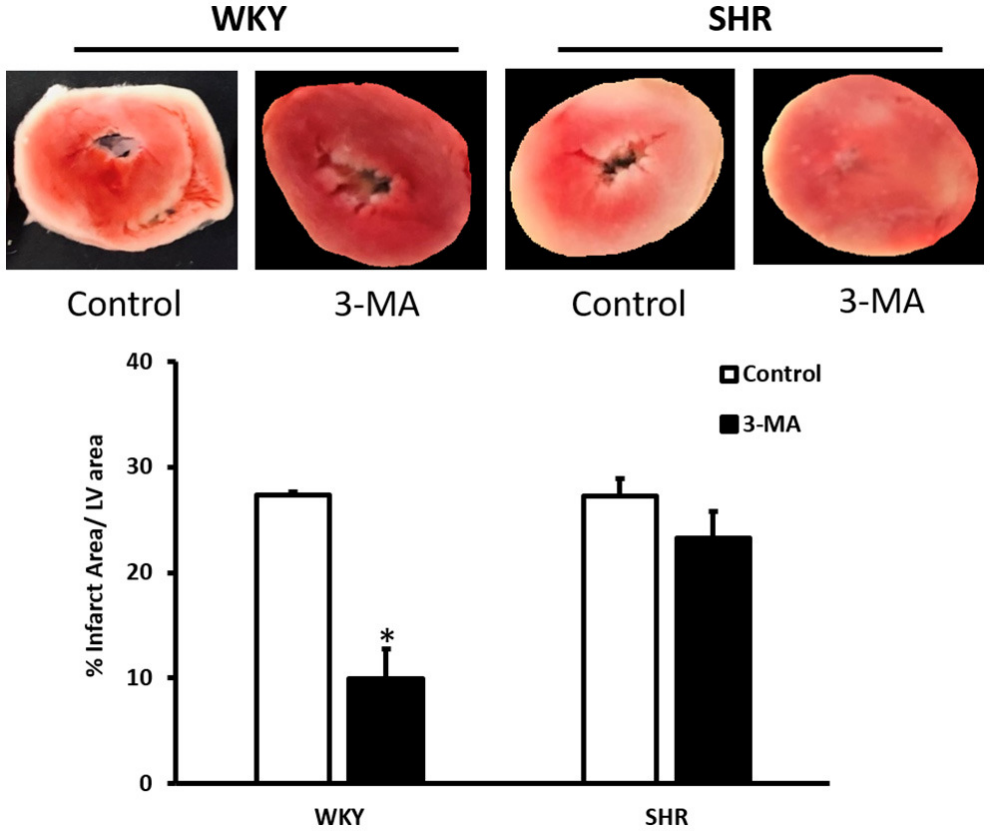
Effect of 3-MA on infarct size in WKY and SHR. TTC staining of heart section from WKY and SHR treated with vehicle control or 3-MA 2 h prior to sacrifice and subjected to I/R injury *ex-vivo*. Data are means ± SEM, *P < 0.001, *n* = 3–8 rats per group.

We have tested the involvement of autophagy in mediating I/R injury in WKY and SHR using autophagy inhibitor 3-MA. Administration of 3-MA *in vivo* significantly (*P* < 0.05) improved cardiac hemodynamics and cardiac contractility in WKY during reperfusion relative to ischemia and relative to untreated controls (Figures 7A–F). Treatment of SHR with 3-MA however, did not result in any significant improvements in the tested parameters at reperfusion compared to ischemia or relative to untreated controls (Figures 7A–F). Consistent with parameters of cardiac physiology, 3-MA treatment significantly (*P* < 0.05) reduced troponin levels in WKY but not in SHR (Table 3). Moreover, 3-MA treatment significantly (*P* < 0.001) reduced the infarct size in WKY but not SHR (Figure 8). This suggests that blockage of autophagy protects WKY against I/R injury, yet it's not sufficient to induce protection in SHR. Protection against myocardial I/R injury in SHR may possibly require the simultaneous activation of multiple cardioprotective effects.

**Table 3:** Effect of blockage of autophagy on troponin release in WKY and SHR.

**Troponin (ng/ml)**				
	**Control**		**3-MA**	
	**Ischemia**	**Reperfusion**	**Ischemia**	**Reperfusion**
**WKY**	0.3 ± 0.1	0.49 ± 0.1	0.62 ± 0.1	0.37 ± 0.042*
**SHR**	0.16 ± 0.02	0.25 ± 0.1	0.15 ± 0.038	0.42 ± 0.1*

*P <0.05 vs. ischemia.

**Figure 9:**
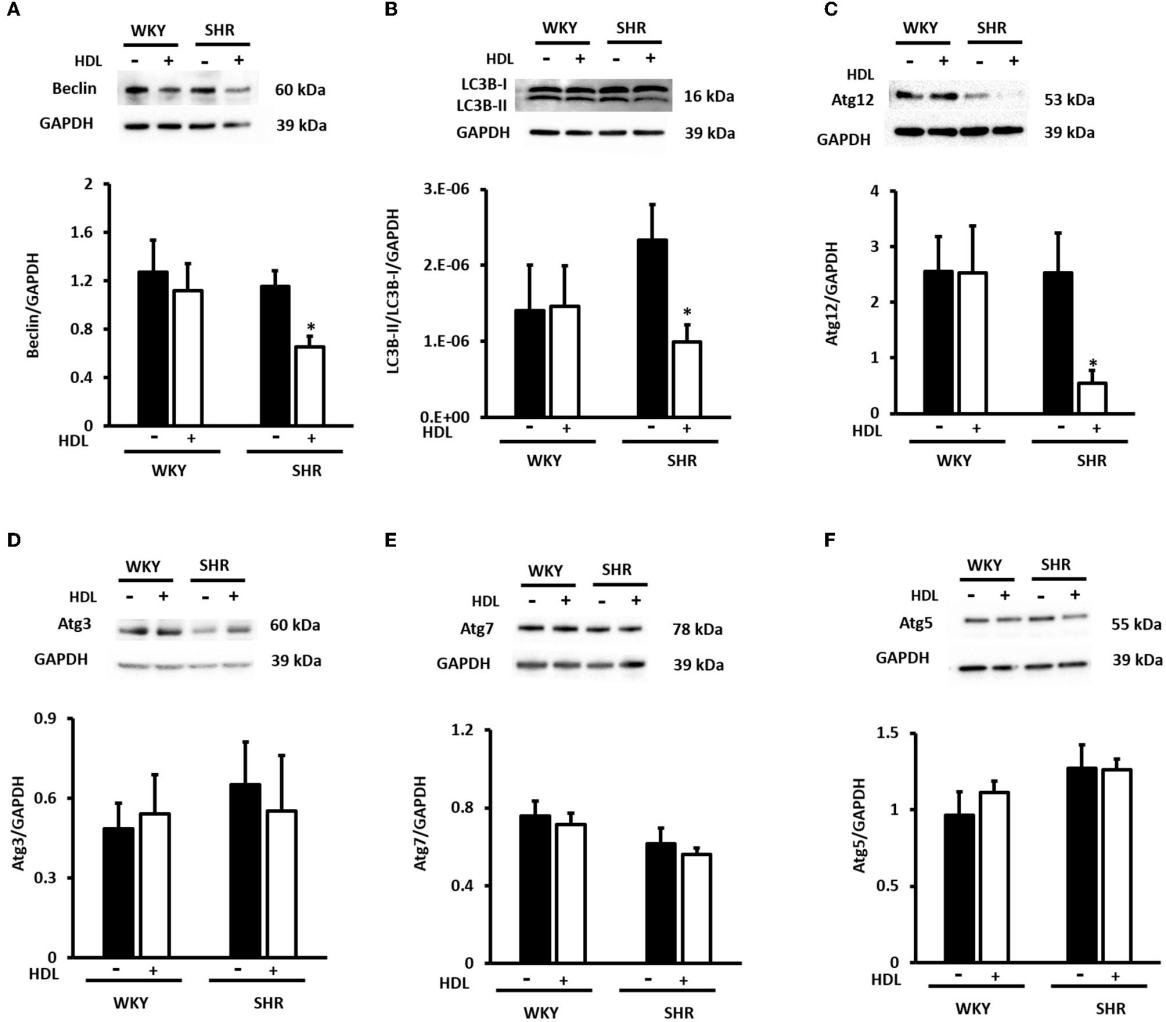
Effect of chronic HDL treatment on the expression of autophagy regulated genes in WKY and SHR. Total heart homogenates were subjected to immunoblotting against beclin, LC3B, Atg-12, Atg-3, Atg-7, Atg-5, and GAPDH as a loading control. Representative images of the relative expression of beclin **(A)**, LC3B-I and II **(B)**, Atg-12 **(C)** Atg-3 **(D)**, Atg-7 **(E)**, and Atg-5 **(F)** in WKY and SHR treated with or without HDL. Data are means ± SEM, *P < 0.05, *n* = 3–8 rats per group.

**Figure 10:**
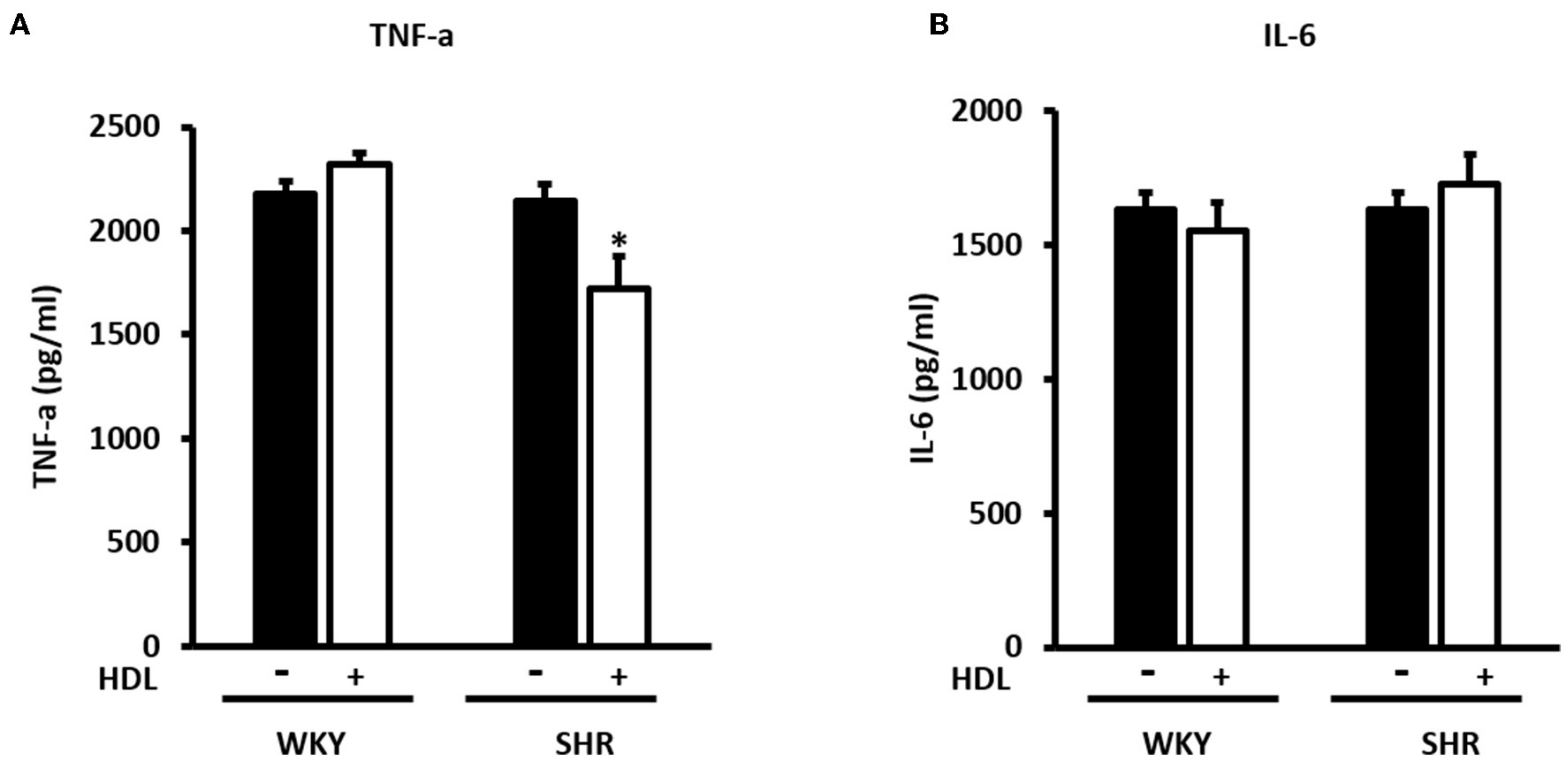
Effect of chronic HDL treatment on inflammatory markers in WKY and SHR. The levels of TNF-α **(A)** and IL-6 **(B)** from hearts treated (chronically) with or without HDL and subjected to I/R injury. Data are means ± SEM, *P < 0.05, *n* = 5–8 rats per group.

We have then investigated the involvement of autophagy in the observed cardioprotective effects of HDL in heart homogenates from WKY and SHR treated with or without HDL. HDL treatment significantly (*P* < 0.05) reduced protein levels of autophagy markers beclin, by 43%, LC3B, by 57%, and Atg-12, by 81.5%, in SHR but not WKY (Figure 9). The expression of other autophagy markers including Atg-3, Atg-5, and Atg-7 was not however significantly altered upon HDL treatment in WKY or SHR (Figure 9). This suggests that HDL reduced autophagy might be a mechanism by which HDL protects against I/R injury in SHR. Furthermore, HDL treatment significantly (*P* < 0.05) reduced TNF-α levels, by about 20%, in SHR but not in WKY (Figure 10A). Nonetheless, HDL treatment did not have a significant effect on IL-6 levels in WKY or SHR (Figure 10B). Together these data suggest that treatment with HDL protects WKY and SHR against ischemic damage, yet distinct protection mechanisms appear to exist in WKY and SHR.

## Discussion

**Figure 11:**
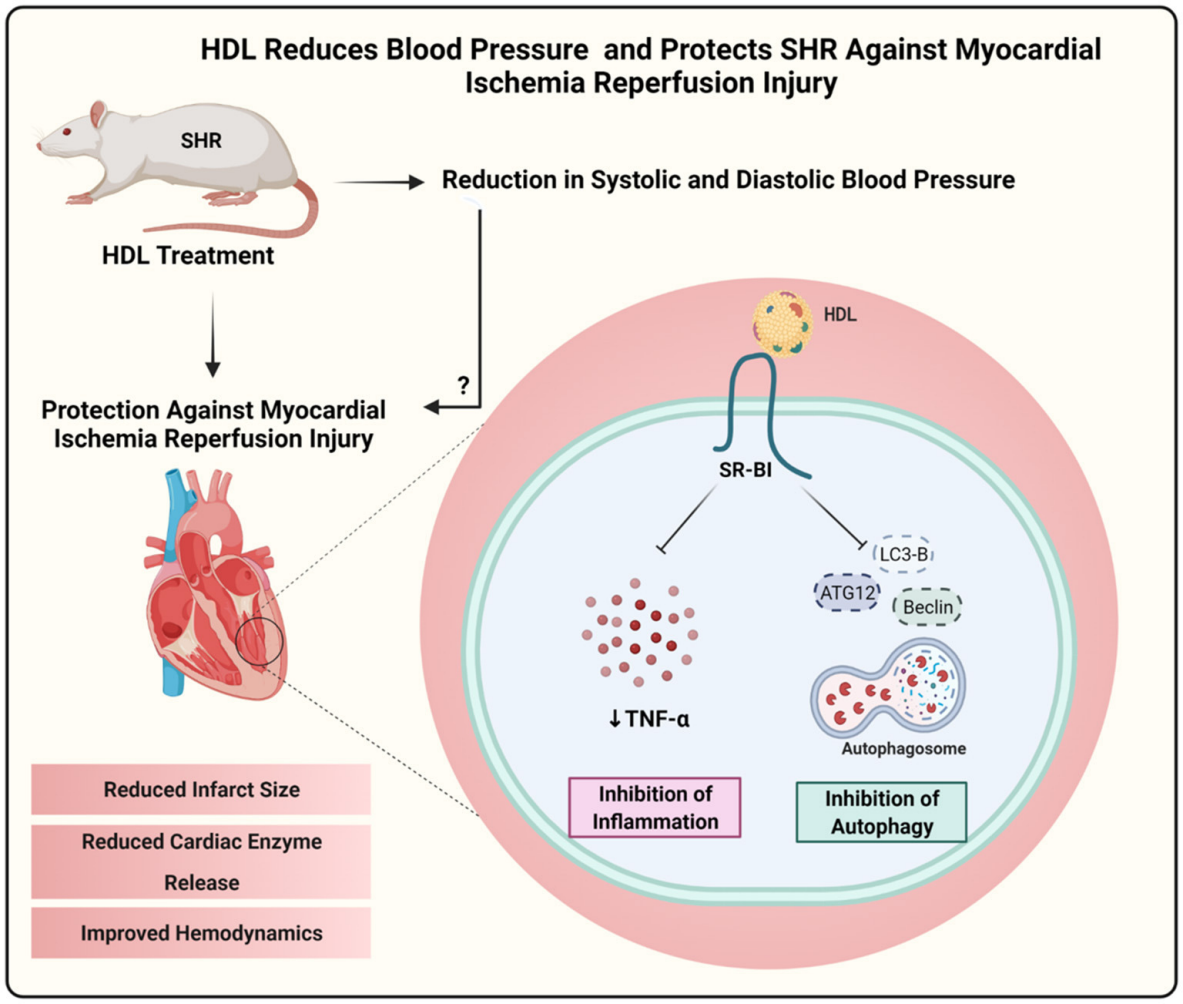
Proposed mechanism of HDL mediated cardiac protection in SHR. Chronic, *in vivo* treatment with HDL reduced blood pressure and protected SHR against myocardial I/R injury. HDL induced cardiac protection required SR-BI and involved attenuation of autophagy and inflammation. Created with Biorender.com.

Hypertension impairs cardiac function (23–26) and alters HDL structure and function (9, 22). HDL protects against I/R injury *in vivo* and *ex vivo*, reviewed in (36). The exact role of HDL in hypertension is however not clearly understood. We have therefore tested the effect HDL on cardiac I/R injury and investigated the possible underlying mechanism(s) in SHR. We demonstrate that HDL treatment reduces SBP and DBP in SHR and protects against cardiac I/R injury in normotensive and hypertensive rats. HDL induced cardiac protection was SR-BI-dependent. Interestingly, the magnitude of HDL mediated protection against I/R injury in WKY and SHR was proportional to the expression levels of cardiac SR-BI. Chronic HDL treatment enhanced cardiac SR-BI expression in SHR and resulted in greater protection against I/R injury in these rats. Chronic HDL treatment reduced cardiac autophagy markers and TNF-α levels in SHR. Our data suggest that in addition to HDL-mediated reduction in SBP and DBP, HDL attenuation of autophagy and inflammation are potential mechanisms by which HDL treatment induces cardiac protection in SHR (Figure 11).

Studies in experimental animals have unanimously shown that HDL protects against cardiac I/R injury in the isolated hearts and *in vivo* (37–39, 44–47). Hearts from hypertensive rodents however, appear to be resistant to cardiac protection induced by multiple agents proven to be protective in normotensive controls (30–34). We have therefore tested if HDL treatment can protect against cardiac I/R injury in the well-established model of hypertension, SHR. Interestingly, HDL infusion at (900 ng/kg/min) for a week significantly reduced SBP and DBP in SHR, however, blood pressure of HDL infused SHR remained significantly higher than untreated WKY. Furthermore, HDL treatment did not change heart to tibia length ratio in WKY or SHR. HDL induced reduction in blood pressure appears to be specific to SHR and was not observed in WKY. A lack of an effect of HDL infusion on blood pressure and heart weight in normotensive controls is consistent with previously reported data (40). HDL mediated reduction in blood pressure in hypertensive rats (Table 1) could be due to reduction in cardiac contractility and cardiac output (48). Alternatively, HDL could enhance nitric oxide production and reduce vascular resistance (49–51). Moreover, HDL could interact reciprocally with the renin-angiotensin system and reduce blood pressure (52, 53). The exact mechanism of blood pressure lowering effect of HDL however, remains to be investigated. Unlike continuous administration of total HDL (our findings), intermittent intravenous administration of reconstituted HDL (rHDL) did not have any significant effects on SBP or cardiac functions in SHR (54). The lack of a blood pressure lowering and cardioprotective effects in response to intermittent rHDL infusion could be attributed to the type, dose, duration or mode of HDL administration.

Furthermore, our data suggest that chronic HDL infusion does not alter plasma total or HDL cholesterol levels in WKY and SHR, possibly suggesting enhanced clearance in response to HDL infusion. Studies involving deletion (55) or overexpression (56) of hepatic SR-BI demonstrated a key role of the receptor in mediating the selective uptake of HDL associated lipids. Overexpression of hepatic SR-BI resulted in a substantial decrease in plasma HDL cholesterol simultaneously with a significant increase in bile cholesterol indicating a role of hepatic SR-BI in RCT (57). Changes in hepatic SR-BI expression levels has been linked to changes in HDL composition (58, 59). Our finding that hepatic SR-BI protein levels are increased in HDL- treated SHR presumably suggests enhanced HDL clearance and supports the maintenance of homeostatic steady state levels of HDL cholesterol. Nonetheless, changes in HDL composition and/or function in response to increased SR-BI expression cannot be excluded (60, 61).

We further demonstrated that chronic HDL treatment protected WKY and, to a greater extent, SHR against cardiac I/R injury. Chronic HDL treatment improved LV pressure, cardiac contractility and hemodynamics at reperfusion relative to ischemia and relative to controls in SHR. Furthermore, chronic HDL treatment reduced cardiac enzyme release and infarct size in SHR. Our finding that HDL treatment protects against myocardial I/R injury in normotensive rats is consistent with previously reported data (37, 39). Nonetheless, to our knowledge, this is the first report that demonstrates a blood pressure lowering and a cardioprotective effect of HDL in hypertensive rodents.

HDL mediated cardiac protection in SHR could be due to the restoration of HDL function and/or composition upon infusion. Alternatively, it could be due to enhanced HDL mediated signaling or both. HDL mediated cardiac protection *via* signaling through SR-BI (62). In WKY, SR-BI blocking antibody significantly reduced but did not completely block the cardioprotective effects of HDL (Figure 5). In SHR however, SR-BI blockage was detrimental. This suggests that in addition to SR-BI, other cardioprotective pathways could also be involved in WKY; the nature of which, however, remains to be investigated. S1P receptor 3 (S1PR3) has been directly implicated in mediating the cardioprotective effects of HDL in an *in vivo* mouse model of myocardial infarction (37). Combined blockage of SR-BI and potential protective pathway(s), possibly S1PR3, may completely abrogate HDL-induced cardiac protection in WKY. The absolute requirement of SR-BI in HDL mediated cardiac protection and the enhanced cardioprotective effects in response to chronic HDL treatment in SHR led us to hypothesize that the enhanced cardiac protection in HDL treated SHR could perhaps be due to enhanced SR-BI expression in these rats. We observed increased cardiac SR-BI expression in SHR in response to chronic treatment with HDL (Figure 6C), consistent with the enhanced protection observed in these rats relative to the normotensive controls (Figure 3). In agreement with these findings, normotensive rats expressed significantly higher levels of cardiac SR-BI in membrane fractions (Figure 6B) but not in total heart homogenates (Figure 6A) and demonstrated an enhanced response to HDL (acute treatment) relative to hypertensive rats (Figure 5). These data not only suggest that SR-BI mediates the protective actions of HDL but it also provide a proof of principle that the magnitude of HDL induced protection is proportional to SR-BI protein levels. Enhanced HDL induced protection is observed in rats expressing greater levels of cardiac SR-BI. Our data further, shed light on differences in cardiac SR-BI localization between WKY and SHR. Despite the fact that no significant differences were observed in SR-BI expression in total heart homogenates from WKY and SHR, significantly greater levels of cardiac SR-BI were localized in the membrane fractions of WKY, implicating enhanced HDL-SR-BI interaction, augmented SR-BI mediated signaling and exacerbated cardiac protection in response to HDL. Moreover, our data highlight the novel upregulation of cardiac SR-BI in response to surgical stress in SHR, revealing a previously undiscovered role of cardiac SR-BI in hypertensive rats subjected to a stress response. The significance of these finding, however, awaits further investigation. Enhanced expression of SR-BI, specifically in the infarcted area, has recently been reported in rats subjected to myocardial infarction induced by left coronary ligation, plausibly suggesting a role of SR-BI in myocardial repair (63). At a cellular level, SR-BI mediated the cardioprotective effects of HDL against necrosis induced by oxygen and glucose deprivation in neonatal mouse and human cardiomyocytes (64). A remarkable role of hepatic SR-BI in correcting cardiac dysfunction has recently been reported in SR-BI KO mice overexpressing hepatic SR-BI (65). Hepatocyte specific transfer of the gene encoding SR-BI restored HDL metabolism and abrogated the detrimental effects on cardiac structure and function in mice lacking SR-BI and subjected to transverse aortic constriction (65). Therefore, in addition to enhanced expression of cardiac SR-BI upon HDL treatment, a role of hepatic SR-BI in HDL mediated protection against cardiac I/R injury in SHR cannot be excluded, at least in part *via* the modulation of HDL metabolism in these rats.

The role of autophagy in cardiac I/R injury appears to be controversial and a double edged sword function of autophagy has been proposed (66, 67). We report that treatment with autophagy inhibitor, 3-MA, protects hearts from normotensive but not hypertensive rats against I/R injury. In line with other reports (68, 69), our data suggest a detrimental role of autophagy in mediating I/R injury and blockage of autophagy is protective, at least, in WKY. The lack of cardiac protection in response to autophagy inhibition in SHR may suggest enhanced deteriorations and impairments in these hearts that blockage of a single pathway, autophagy, is not sufficient to induce protection. Consistent with these findings, SHR were resistant to cardiac protection induced by helium, erythropoietin and captopril (33–35).

HDL inhibited mechanical stress induced cardiac hypertrophy and autophagy in cultured cardiomyocytes and *in vivo, via* the downregulation of cardiac angiotensin receptor 1 and signaling *via* the PI3K/Akt pathway (70). Furthermore, HDL inhibited autophagic responses in endothelial cells treated with oxLDL (71). On contrary, glycated HDL increased the expression of autophagic proteins beclin, LC3B and Atg-5 in macrophages (72). Our data indicate that chronic HDL treatment reduces the expression levels of autophagy markers, beclin, LC3B and Atg-12 in SHR hearts subjected to I/R injury. Nevertheless, this was not observed in WKY treated with HDL. This suggests that HDL mediated reduction is autophagy might be one protective mechanism by which HDL treatment protects against cardiac I/R injury in hypertensive rats. We further report that chronic HDL treatment reduces cardiac TNF-α levels in SHR. This however, was not observed in WKY. Reciprocal interaction between autophagy and proinflammatory cytokines has been reported. TNF-α treatment was shown to enhance autophagy (73), while 3-MA mediated inhibition of autophagy upregulated TNF-α and IL-6 levels in immortalized bone marrow derived macrophages (74). Our findings indicate that chronic HDL treatment induces cardiac protection in SHR *via* multiple mechanisms, including attenuation of autophagy and inflammation. It remains to be tested however whether HDL attenuates autophagy *via* its anti-inflammatory effects or reduces inflammation by virtue of its effects on autophagy. However, while HDL was protective in WKY, HDL mediated reduction in autophagy and inflammation does not seem to play a significant role in normotensive rats, treated with HDL at least under these experimental conditions. Alternative protection mechanisms may operate in WKY, nonetheless the effects of HDL on inflammation and autophagy in WKY cannot be completely excluded. HDL attenuation of inflammation and autophagy could be involved in HDL mediated protection in WKY however, it might operate at different kinetics and involve markers other than what we used in this study.

The strengths of this study include reporting a novel anti-hypertensive effect of HDL in SHR. In addition, this study elucidated an interesting role of cardiac SR-BI in HDL-mediated protection against I/R injury in normotensive and hypertensive rats whereby the magnitude of HDL induced cardiac protection was directly proportional to the expression levels of cardiac SR-BI. Furthermore, our study unraveled differences in the mechanism(s) by which HDL protected against myocardial injury in WKY and SHR. Nonetheless, our study has some limitations related to the dose, duration and timing of the HDL treatment. It's not clear if HDL administration to younger, perhaps 4 week old, SHR can prevent the development of hypertension or if higher doses of HDL or longer periods of administration will result in further reduction in BP. Male SHR exhibited significantly higher SBP values (*P* < 0.05) at 12-week of age and thereafter than female SHR (75). We demonstrate that HDL treatment reduces SBP and protects against myocardial I/R injury in male SHR. Nonetheless, it not clear if HDL is equally protective in female SHR. Moreover, the study did not investigate if HDL effects will permanently last or be temporarily effective during the treatment period. Flow up studies are required to address these limitations and further investigate the molecular mechanisms of HDL induced cardiac protection.

In conclusion this study shed light on a novel role of HDL in hypertension. HDL treatment reduced blood pressure and protected hypertensive rats against cardiac I/R injury. We demonstrate that chronic HDL treatment reduces blood pressure in SHR and protects against cardiac I/R injury. HDL induced protection appears to involve signaling *via* SR-BI and attenuation of cardiac autophagy and inflammation.

## Data Availability Statement

The original contributions presented in the study are included in the article/Supplementary Material, further inquiries can be directed to the corresponding author/s.

## Ethics Statement

The animal study was reviewed and approved by Health Sciences Research Ethics Committee Kuwait University.

## Author Contributions

AA-J obtained the funding for the study and drafted the manuscript. AA-J and FB designed and supervised conducting the experiments, analyzed the data, edited and revised the manuscript. Both authors contributed to the article and approved the submitted version.

## Funding

This project was funded by the Research Administration at Kuwait University. Grant No. MB03/16.

## Conflict of Interest

The authors declare that the research was conducted in the absence of any commercial or financial relationships that could be construed as a potential conflict of interest.

## Publisher's Note

All claims expressed in this article are solely those of the authors and do not necessarily represent those of their affiliated organizations, or those of the publisher, the editors and the reviewers. Any product that may be evaluated in this article, or claim that may be made by its manufacturer, is not guaranteed or endorsed by the publisher.

## Supplementary Material

Supplementary Figure 1 Heart to tibia length ratios. Herat weights of WKY and SHR rats implanted with vehicle or HDL containing pumps were expressed relative to tibia lengths. Data are mean ± SEM, *n* = 7–10 rats per group.
