# Supplementary figures and images for: High Density Lipoprotein Reduces Blood Pressure and Protects Spontaneously Hypertensive Rats Against Myocardial Ischemia-Reperfusion Injury in an SR-BI Dependent Manner
Source: Front Cardiovasc Med. 2022 Mar 21;9:825310. doi: 10.3389/fcvm.2022.825310 (PMC8977778; doi:10.3389/fcvm.2022.825310)

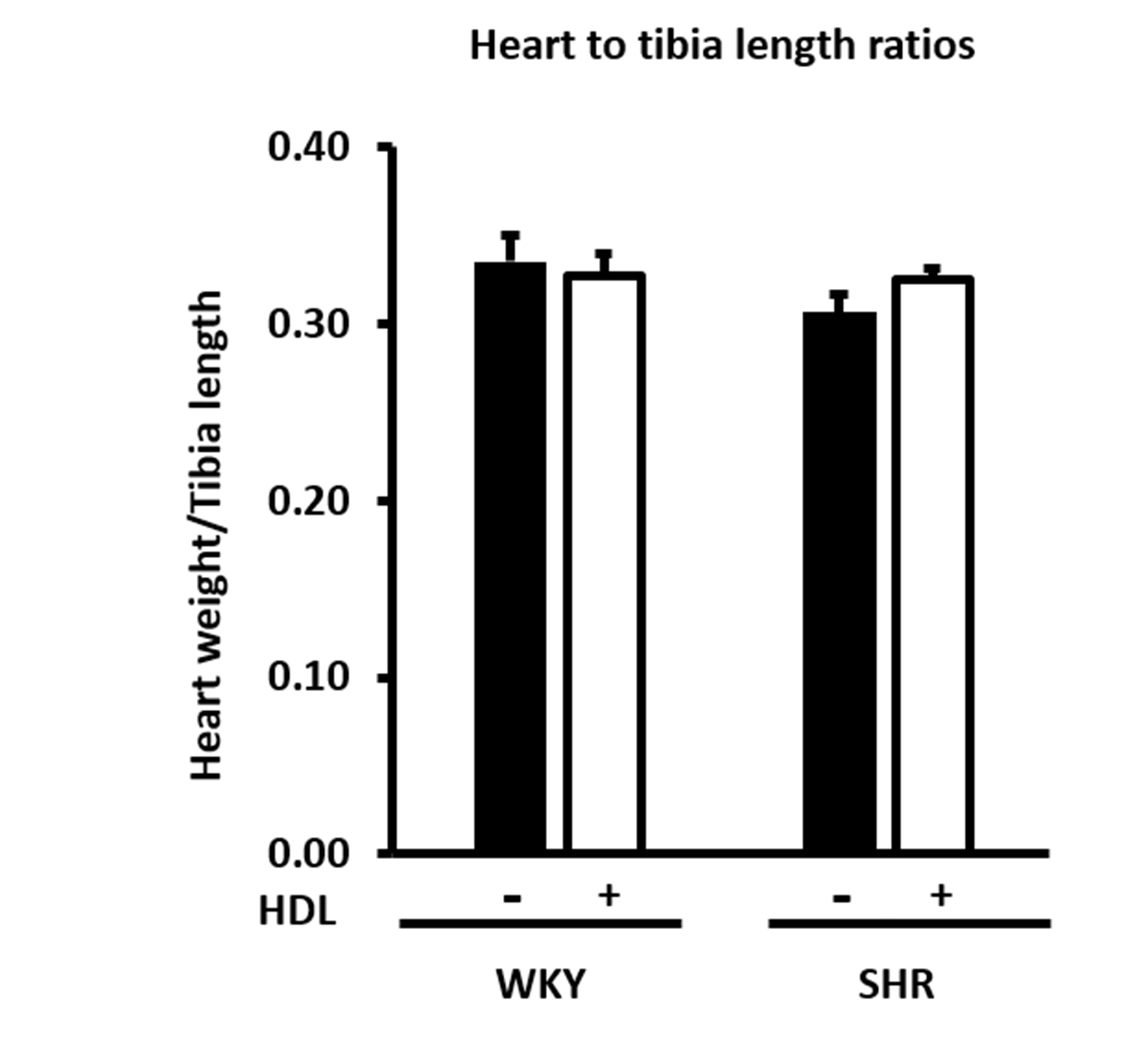

Supplement: Supplementary Figure 1 — Heart to tibia length ratios. Herat weights of WKY and SHR rats implanted with vehicle or HDL containing pumps were expressed relative to tibia lengths. Data are mean ± SEM, n = 7–10 rats per group. [file Image_1.tif]
